# Deregulation of Plasma microRNA Expression in a *TARDBP*-ALS Family

**DOI:** 10.3390/biom13040706

**Published:** 2023-04-21

**Authors:** Paola Ruffo, Stefania Catalano, Vincenzo La Bella, Francesca Luisa Conforti

**Affiliations:** 1 Medical Genetics Laboratory, Department of Pharmacy, Health and Nutritional Sciences, University of Calabria, 87036 Rende, Italy; paola.ruffo@unical.it; 2 Laboratory of Neurogenetics, National Institute on Aging, National Institutes of Health, Bethesda, MD 20892, USA; 3 Department of Pharmacy, Health and Nutritional Sciences, University of Calabria, 87036 Rende, Italy; stefania.catalano@unical.it; 4 ALS Clinical Research Centre and Laboratory of Neurochemistry, Department of Experimental Biomedicine and Clinical Neurosciences, University of Palermo, 90133 Palermo, Italy; vincenzo.labella@unipa.it

**Keywords:** amyotrophic lateral sclerosis, microRNA, plasma, different expression, mutation carriers, *TARDBP*

## Abstract

TDP-43 intracellular aggregates are a pathogenic sign of most amyotrophic lateral sclerosis (ALS) cases. Familial ALS, brought on by *TARDBP* gene mutations, emphasizes the relevance of this altered protein in pathophysiology. Growing evidence suggests a role for dysregulated microRNA (miRNA) in ALS disease. Furthermore, several studies showed that miRNAs are highly stable in various biological fluids (CSF, blood, plasma, and serum), and they are expressed differentially by comparing ALS patients and controls. In 2011, our research group discovered a rare mutation in a *TARDBP* gene (G376D) in a large ALS Apulian family with affected members exhibiting a rapidly progressing disease. To identify potential non-invasive biomarkers of preclinical and clinical progression in the *TARDBP*-ALS family, we assessed the expression levels of plasma microRNAs in affected patients (n = 7) and asymptomatic mutation carriers (n = 7) compared with healthy controls (n = 13). Applying qPCR, we investigate 10 miRNAs that bind TDP-43 in vitro during their biogenesis or in their mature form, and the other nine are known to be deregulated in the disease. We highlight the potential of miR-132-5p, miR-132-3p, miR-124-3p, and miR-133a-3p expression levels in plasma as biomarkers of preclinical progression for G376D-*TARDBP*-associated ALS. Our research strongly supports the potential of plasma miRNAs as biomarkers for performing predictive diagnostics and identifying new therapeutic targets.

## 1. Introduction

Amyotrophic lateral sclerosis (ALS, also known as Lou Gehrig’s disease) is a progressive degenerative disease affecting motor neurons (MNs) with a multifactorial etiology in which both environmental and genetic variables play a crucial role [1,2]. ALS appears to be a heterogeneous disease, not only regarding the age of onset or clinical phenotype but, most importantly, related to its rate of progression [3,4]. The disease occurs in sporadic (sALS, about 90%) and familial forms (fALS, about 10%), with an estimated incidence of 0.4 and 1.8 per 100,000 inhabitants [5]. About 40–55% of cases with a familial background are due to pathogenic mutations in genes coding for Superoxide Dismutase 1 (*SOD1*) [6], Fused in Sarcoma (*FUS*) [7], TAR DNA-binding protein 43 (*TARDBP*) [8] and a hexanucleotide expansion on chromosome 9 in Open Reading Frame 72 (*C9Orf72*) [9].

Among these, the role of *TARDBP* as a component of the nuclear complexes of Drosha and cytoplasmic Dicer is now known. Specifically, *TARDBP* facilitates the binding of the Drosha complex to a subset of pri-miRNAs resulting in cleavage into pre-miRNAs, while interaction with Dicer facilitates the formation of a specific subset of pre-miRNAs via direct binding to the terminal loops. Since it promotes the biogenesis of miRNAs, the dysregulation of *TARDBP* activity may be associated with the pathogenesis of ALS and could affect the expression levels of miRNAs [10].

The actual diagnosis of fALS and sALS is mainly based on the EI Escorial, Awaji, and Gold Coast criteria [11,12,13]. Only recently, CSF and blood biomarkers have emerged as useful tools for diagnosis and disease progression/survival [14]. Furthermore, neurofilaments biomarkers have been used to identify premanifest mutation carriers approaching phenoconversion [15]. Indeed, it is necessary to look for new clinical biomarkers for the early detection of ALS, for the stratification of cases based on phenotype, and for the selection of the most effective therapy approach. In this scenario, miRNAs seem to be interesting biomarker molecules in ALS.

miRNAs are short endogenous sequences (20–22 nucleotides) of single-stranded non-coding RNA involved in finely controlling gene expression. They are tissue-specific, highly correlated with pathology, and can be isolated and measured with minimal invasiveness when isolated from human biological fluids [16]. Several studies highlight the role of miRNA in ALS pathology by describing dysregulated miRNAs in various biological fluids (CSF, blood, plasma, and serum) in ALS patients compared to healthy controls [17]. It has been shown that miRNAs specifically produced in motor neurons are present in the plasma of ALS patients and are correlated with disease severity [18]. MiRNAs have several intrinsic characteristics that make them attractive as biomarkers. It has been shown that miRNAs have a high specificity [19], are remarkably stable [20], and can be used to “capture” changes in source cells, including neurons [16,21].

In 2011, our research group identified a new TARDBP mutation, the G376D, in a woman with progressive upper-limb weakness [22]. The reconstruction of the genealogic tree led us to a large collection of DNA, plasma, and fibroblasts from family members, either affected or not. The segregation analysis revealed a dominant pattern of transmission, although the penetrance appears to be incomplete.

In 2013, Czell and colleagues performed a genetic screening on the Swiss population and described an Italian patient with the same mutation [23]. In 2018, another case carrying the G376D mutation was reported in an Asian familial ALS individual [24].

In this context, we sought to study several miRNA expressions in the ALS family carrying the G376D *TARDBP* mutation. Specifically, in this project, we will investigate the differential circulating miRNAs in plasma from healthy individuals, asymptomatic mutation carriers, and affected patients, all belonging to a large Apulian family.

## 2. Materials and Methods

### 2.1. Patient Cohorts and Ethics Statements

The patient cohort investigated in this study includes 7 symptomatic patients, 7 asymptomatic mutation carriers, and 13 healthy controls, all belonging to a large Apulian family carrying a G376D *TARDBP* ALS-causing mutation. To our knowledge, this mutation has been identified only in this family, among Caucasians. All the participants recruited had signed an informed consent form. All subjects described in the manuscript, i.e., healthy carriers (asymptomatic subjects), the symptomatic carriers (i.e., the patients), and the healthy non-carriers, were submitted to a short interview concerning the medical history and actual signs/symptoms of ALS and a focused neuromuscular examination as well. The examiners were two expert neurologists (one of the two is the author VLB) who were unaware of the final results of the genetic testing. All data were recorded in the database of the ALS Clinical Research Center of Palermo, Italy. Subjects were not submitted to EMG/ENG examination and/or neuropsychological testing. None of the healthy mutation carriers and healthy controls showed signs/symptoms related to motor neuron disease. According to Benatar M et al., 2022 [15], our asymptomatic mutation carriers were all in a clinically silent pre-manifest state.

Peripheral blood samples were collected according to standard procedures in test tubes containing EDTA and centrifuged to allow separation of the plasma from the cellular component. Then, plasma was transferred to microtubes and centrifuged again at 12,000 rpm for 10 min to eliminate additional cellular residues.

### 2.2. RNA Isolation

Total circulating RNA, including small RNAs, was isolated from the plasma of both asymptomatic and symptomatic mutation carriers and of healthy non-carriers, using MagMAX™ mirVana™ Total RNA Isolation Kit (Applied Biosystem, Thermo Fisher Scientific, Foster City, CA, USA). RNA was isolated in each case from 200 μL of plasma following a methodology as specified by the manufacturer. microRNA was quantitated by Invitrogen™ Qubit™ 3.0 Fluorometer (Invitrogen, Thermo Fisher Scientific, Waltham, MA, USA). Cel-miR-39-3p, a synthetic miR-39-3p of Caenorhabditis elegant, was used as an RNA spike-in to normalize the gene expression analysis and to adjust different RNA isolation and reverse transcription efficiencies.

### 2.3. Reverse Transcription and Quantitative PCR

Equal amounts (50 ng) of miRNA were used for reverse transcription (RT) using the TaqMan miRNA Reverse Transcription Kit. Amplification was performed by quantitative PCR (qPCR) on the QuantStudio™ 3 Real-Time PCR System (Applied Biosystem by Thermo Fisher Scientific) using TaqMan MicroRNA Assays (Applied Biosystem by Thermo Fisher Scientific) of selected miRNAs and endogenous controls (Supplementary Table S1). Each sample was analyzed in triplicate, and the expression was calculated using the 2^−ΔΔCt^ method [25]. Furthermore, plasma miRNA levels were normalized against an exogenous oligonucleotide (cel-miR-39-3p) and two endogenous (hsa-miR-191-5p and hsa-miR-93-5p) that were unchanged in the assay.

### 2.4. Statistical Analysis

All analyses were carried out using GraphPad Prism software version 7.0 (GraphPad Software, San Diego, CA, USA). Variables were expressed as median, with an interquartile interval between Q1 and Q3 values (IQR). The non-parametric data were analyzed with the Mann–Whitney rank sum test. Categorial data were evaluated using Fisher’s exact test. We adopted the Kruskal–Wallis ANOVA on ranks to compare data of multiple groups: symptomatic compared to healthy samples, asymptomatic about non-affected cases, and symptomatic contrasted with asymptomatic subjects. The data obtained were statistically analyzed using 1-way ANOVA and the pair-wise Tukey (HSD) test to study the difference between group mean values (α = 0.05). *p*-values less than 0.05 were considered statistically significant.

## 3. Results

In this study, we analyzed the expression of 19 miRNAs in the asymptomatic and symptomatic carriers of a large ALS family with an aggressive ALS-causing *TARDBP*-G376D mutation. Healthy non-carrier subjects belonging to the same family were used as controls.

The detailed characteristics of all family subjects are summarized in Table 1.

The median age-at-onset in the symptomatic mutation carriers was 50 years (IQR = 37–57), which appears primarily younger than the age-at-onset of the sporadic ALS in large case series [26]. Conversely, it aligns with the age at the onset of genetic ALS with an autosomal dominant transmission pattern and relatively high penetrance. Furthermore, none of the symptomatic mutation carriers was older than 57 at the disease onset. In contrast, 30% of the asymptomatic mutation carriers were older than 70 at the time of the blood draw. This suggests that, at least in this family, beyond a certain age (we suggest seventy years as a cut-off value), the likelihood of developing the disease in the asymptomatic mutation carriers might decrease dramatically.

The proportion of the spinal-onset vs. bulbar-onset in the symptomatic mutation carrier cohort was 70% vs. 30%, respectively. Again, this data suggests that the G376 mutation does not show site-of-onset preference, even in a small cohort. Finally, the relative mild disability at diagnosis in the symptomatic cohort underlines that the diagnostic delay in these patients was relatively short, evidence supported by the high suspicion index in members of this family with a disease-causing mutation.

We investigated and validated miRNAs (n = 10) binding TDP-43 in vitro during their biogenesis or in their mature form [10,27], already studied by Freischmidt et al. 2013 [28], and miRNAs (n = 9) known to be deregulated in post-mortem tissues, specifically cell lines and several circulating fluids [17] in 7 symptomatic patients, 7 asymptomatic mutation carriers and 13 healthy controls (Supplementary Table S1). Of these selected miRNAs, four probes (has-miR-663a, has-miR-155-3p, has-miR-218-5p, has-miR-338-5p) were not confirmed due to lack of detection in the plasma matrix.

**Figure 1:**
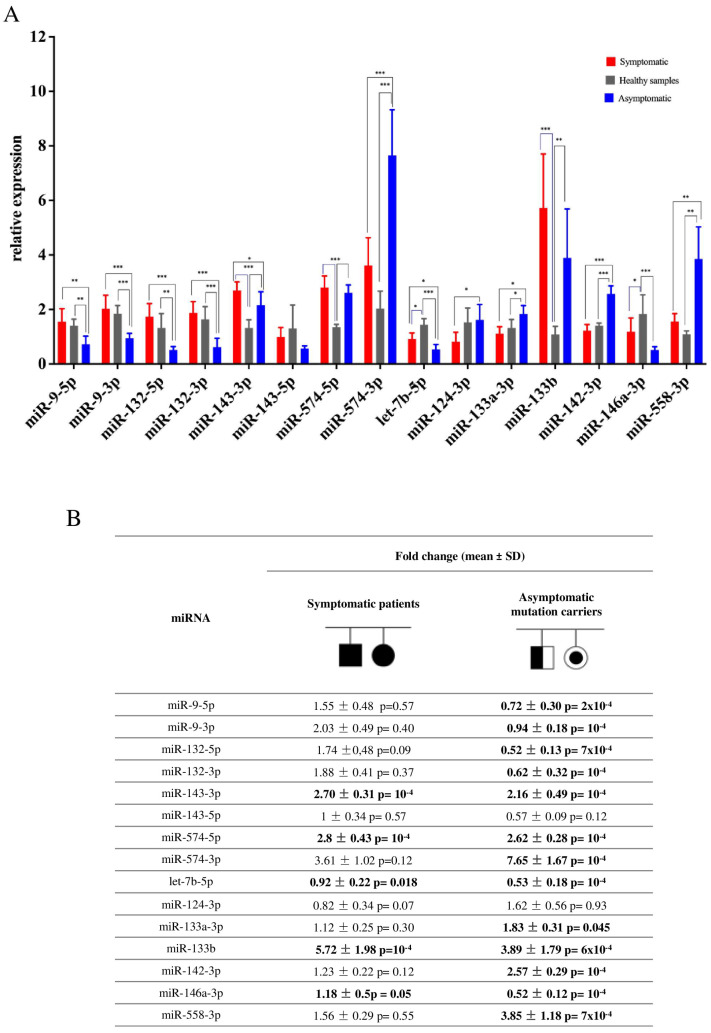
(**A**) miRNA dysregulation in plasma of ALS family subjects with *TARDBP*-G376D mutation compared to healthy control samples. *: *p* < 0.05: **: *p* < 0.01. ***: *p* < 0.001; (**B**) relative miRNA expression in a *TARDBP*-G376G family compared to healthy family members. miR = miRNA; statistically significant differences are highlighted in bold. SD = standard deviation.

To distinguish miRNA tags investigated, we questioned DIANA TarBase v.8 [29]. We identified only three miRNAs that target *TARDBP* (Supplementary Table S2). We observed a significant plasma upregulation of miR-143-3p, -574-5p, and -133b miRNAs comparing affected patients with healthy controls while let-7b-5p ad miR-146a-3p were downregulated (Figure 1A,B). Furthermore, we observed marked deregulation of our selected miRNAs when comparing asymptomatic mutation carriers with healthy controls (Figure 1A,B).

Relative expression of miRNA in plasma is measured with qPCR. Normalization was performed relative to the spiked-in C. elegans miRNA (Cel-miR-39-3p) and two endogenous miRNAs (hsa-miR-191-5p and hsa-miR-93-5p), and to the mean expression of the respective miRNA in the control group. Bars indicate mean standards deviation.; n = 7 symptomatic patients (P), n = 7 asymptomatic mutation carriers (C), and n = 13 healthy controls (HC). In plasma samples of P, we found a significant increase in miR-143-3p, miR-574-5p, and miR-133b. Only let-7b-5p and miR-146a-3p showed a significant decrease. The remaining miRNA showed no significant changes in serum samples of P compared to HC. Furthermore, in the plasma of the C, we found a significant increase of miR-143-3p, miR-574-5p, miR-574-3p, miR-133a-3p, miR-133b, miR-142-3p, and miR-58-3p. On the contrary, miR-9-5p, miR-9-3p, miR-132-5p, miR-132-3p, let-7b-5p, and miR-146a-3p appears significantly decreased. Only miR-miR-143-5p and miR-124-3p showed no significant changes in plasma samples of C compared to HC.

In our analysis, just one subject showed a very early age of onset (37 years old) compared to the rest of the affected members of the same family and compared to the disease average (Table 1). Furthermore, this patient was characterized by a very aggressive disease with a highly rapid course. The expression levels of the plasma miRNAs analyzed were found to be altered compared to the other affected family members, although the methods of analysis used are the same (Supplementary Figure S1). MiRNA folds change values of the subject are represented in Supplementary Table S3. Given the wide disparity in the values of this patient compared to the other family-affected samples, the relative miRNA expression was not included in the 2^−ΔΔCt^ method analysis and Figure 1A,B.

## 4. Discussion

The present study aimed to uncover circulating biomarkers by analyzing plasma miRNA expression levels in a large ALS family (7 symptomatic patients, 7 asymptomatic mutation carriers, and 13 healthy controls) characterized by a rare mutation in the TARDBP gene. This study focused on determining which miRNA could be involved in protecting or, conversely, predisposing to the development of the disease in subjects carrying the mutated gene. The results of the present study showed a specific miRNA differential expression in the symptomatic and asymptomatic mutation carriers compared to healthy family members, underlining a probable peripheral signature capable of stratifying the disease progression. The asymptomatic mutation carriers’ study, from the presymptomatic state across phenoconversion and clinically expressed ALS, provides a unique opportunity to observe prospectively and shed light on the earliest clinical and molecular features of neurodegeneration.

Freischmidt et al. 2013, were the first to undertake studies identifying potential miRNA-based biomarkers in the serum of ALS patients and asymptomatic mutation carriers [28,30,31]. To the best of our knowledge, the present work is the first to investigate the deregulation of circulating miRNAs in ALS familiar forms with incomplete penetrance, focusing on a rare *TARDBP* mutation in addition to supplying a serum miRNA signature that may contribute to the assessment of preclinical progression for ALS.

A prominent finding of our study is the plasma deregulation of both miR-132-3p and 286 miR-132-5p, capable of differentiating between symptomatic and asymptomatic mutation 287 carriers. This miRNA has repeatedly been implicated in neuronal development and synaptogenesis [32] and could reflect the peripheral pathophysiological mechanisms mainly associated with neurodegeneration. Although miR-132 is known to target *TARDBP*, few articles describe this interplay’s consequences. Karginov FV et al. 2013 is the only work that highlights this interaction tested on a renal cell line [33], which needs further validation. The involvement of miR-132 at the neuronal level, the knowledge of *TARDBP* as a potential target, and serum deregulation could allow the identification of this miRNA as a predictive peripheral marker for ALS disease.

Of interest is the increased peripheral expression of miR-132 and miR-9 in both strands in symptomatic subjects compared to healthy controls (Figure 1B). Although the expression values of these four miRNAs (miR-9-5p, *p* = 0.57; miR-9-3p, *p* = 0.40; miR-132-5p, *p* = 0.09; miR-132-3p, *p* = 0.37) were statistically non-significant in our study, these seem to follow the same trend as neurofilament light chains (NfL): a validated biofluid marker of neuroaxonal damage [34]. Several studies have shown that NfL levels are increased in ALS patients compared to healthy controls and correlate with clinical disease severity measures [35].

miR-9 is known to carry out many functions in nervous system development but does not bind TDP-43. Conversely, *FUS* regulates the biogenesis of miR-9, which subsequently directly binds the FUS protein [36]. This is an evolutionary-conserved brain-enriched miRNA and is the most highly expressed in the nervous system [37]. Both miR-9-5p and -3p have been associated with maintaining neuronal cell and synaptic plasticity [37], resulting in important factors in neurogenesis regulation [38]. The significant upregulation of miR-9-5p and miR-9-3p in ALS symptomatic patients compared to asymptomatic mutation carriers can be considered useful for considering this miRNA as a biomarker for disease progression. In agreement with the literature, the increased expression of miR-9-5p could reflect neuronal injury and cell death [37]. On the other hand, there is little information about miR-9-3p. Sim et al., 2016 have demonstrated that the expression levels of this miRNA are decreased in neurodegenerative diseases such as Huntington’s and Alzheimer’s disease [39]. Although miR-9 appears to be related to the presence of mutations in the *FUS* gene [36], we find a deregulation that could be representative of the disease state. Further studies should be led to better understand the functions of this miRNA and the probable relationship with another gene involved in RNA metabolism such as *TARDBP*.

Another miRNA that targets *TARDBP* is miR-124-3p [40]. This appears to be deregulated exclusively by comparing symptomatic and asymptomatic mutation carriers. miR-124 is undoubtedly involved in ALS through the inflammatory reaction [41], and it is also considered a neuron-specific miRNA because it is mainly expressed in neuronal cells [42]. Based on this knowledge and our result, we could hypothesize that miR-124 levels could be directly related to disease status, making it possible to identify a presymptomatic state of disease.

A trend can be shown for miR-133a-3p and miR-142-3p, which appear to be significantly upregulated in asymptomatic mutation carriers. Overexpression of miR-133a correlated with a better prognosis, and a slower course of disease [21], and serum/plasma levels of miR-133a can predict disease course and severity. In agreement with the literature, our results suggest that miR-133a-3p could be a possible biomarker of disease progression. It is interesting to underline how miR-574-3p, miR-133a-3p, and miR-558-3p have decreased expression in symptomatic patients. This data should be more extensively addressed to understand if it involved a construct downregulation or a silencing mechanism.

On the other hand, miR-142-3p was predicted to target the expression of TDP-43, and in a study conducted by Matamala et al., 2018 an association between this miRNA expression level and disease progression was demonstrated [43]. However, our findings differ from the preceding results: miR-142-3p expression levels appear upregulated in asymptomatic mutation carriers who, therefore, do not exhibit the clinical phenotype of the disease. The results could seem conflicting due to (i) the small number of available samples, (ii) the different cohort analyzed characterized by a familial form of the disease, and (iii) the presence of a precise mutation.

Of particular interest is the differential expression of miR-574-3p which appears to be upregulated in asymptomatic mutation carriers compared to affected patients. This miRNA is known to be a critical player in tumorigenesis [44,45], but there is no clear relationship with neurodegenerative disorders. However, Kawahara et al. 2012, showed that cytoplasmic TDP-43 facilitates the binding of the Dicer complex to a subset of pre-miRNAs and thus promotes the production of mature miRNAs. Based on these reports, we hypothesize that miR-574-3p is specifically deregulated in correlation to a mutation of the *TARDPB* gene, and its overexpression could be correlated to a non-damaging effect against the disease. Given the limited information regarding the -3p arm, it would be interesting to further investigate its mechanism of action in neurodegeneration and ALS.

Our results are encouraging, but our study has some limitations. First, although the present work is based on the analysis of asymptomatic and symptomatic ALS individuals belonging to the same family, the number of samples analyzed to candidate miRNAs as peripheral biomarkers are small. Second, the absence of validation in other tissues or of a replication cohort means that further studies in independent cohorts are needed to confirm our findings. Finally, the absence of multiple collections of biological samples at different stages of disease progression for each symptomatic ALS individual does not allow for any definitive conclusion on the correlation between plasma miRNAs and disease evolution. Our preliminary data could be broadened using high-throughput methods to investigate more constructs and study the interactions with ALS genes.

## 5. Conclusions

The present work represents the first study evaluating plasma miRNAs signature, in asymptomatic mutation carriers and symptomatic *TARDBP* individuals belonging to a large family, as progression biomarkers for ALS. We highlighted the potential of miR- 132-5p, miR-132-3p, miR-124-3p, and miR-133a-3p expression levels in plasma as biomarkers of preclinical progression for G376D-*TARDBP*-associated ALS. In this context, our work could establish a basis for promoting the use of plasma miRNAs, in association with other biomarkers, to perform a predictive diagnosis and discover novel therapeutic targets.

## Figures and Tables

**Table 1:** Clinical and demographic characteristics of the members of the G376D *TARDBP* Apulian family were included in this study. Data are expressed as median with interquartile intervals Q1–Q3.

Variable	SC (n = 7)	AC (n = 7)	HC (n = 13)	*p*
Age ^$^ at blood drawing	52 (42–60)	42 (33–73)	52 (44–76)	0.47 *
Age ^$^ at onset	50 (37–57)	N.A.	N.A.	
Sex (M/F)	1.33	2.5	0.85	0.19 **
Onset Spinal, n (%) Bulbar, n (%)	5 (71) 2(29)	N.A.	N.A.	
ALSFRS-R ^a^	44 (41–45)	N.A.	N.A.	

SC, symptomatic mutation carriers; AC, asymptomatic mutation carriers; HC, healthy non-carrier controls. ^$^ Age is expressed in years; ^a^ evaluation at diagnosis; * Mann–Whitney Rank Sum Test; ** chi-square; N.A.: not applicable.

## Data Availability

The data presented in this study are available in results and supplementary material sections.

## Supplementary Materials

The following supporting information can be downloaded at: https://www.mdpi.com/article/10.3390/biom13040706/s1, Figure S1: Serum miRNA deregulation values of one sample compared to affected subjects from the same family; Table S1: List of miRNAs analyzed with relative assay identification (ID) and mature miRNA sequence.; Table S2: List of miRNAs targeting *TARDBP* (References [33,40,46,47] are cited in the supplementary materials); Table S3: List of serum miRNA values for one specific affected patient normalized against one exogenous (cel-miR-39) and two endogenous (hsa-miR-191-5p and hsa-miR-93-5p) controls.

## References

[1] Mejzini R., Flynn L.L., Pitout I.L., Fletcher S., Wilton S.D., Akkari P.A. (2019). ALS Genetics, Mechanisms, and Therapeutics: Where Are We Now?. Front. Neurosci..

[2] Mead R.J., Shan N., Reiser H.J., Marshall F., Shaw P.J. (2023). Amyotrophic lateral sclerosis: A neurodegenerative disorder poised for successful therapeutic translation. Nat. Rev. Drug Discov..

[3] Ungaro C., Sprovieri T., Morello G., Perrone B., Spampinato A.G., Simone I.L., Trojsi F., Monsurro M.R., Spataro R., La Bella V. (2021). Genetic investigation of amyotrophic lateral sclerosis patients in south Italy: A two-decade analysis. Neurobiol. Aging.

[4] Goyal N.A., Berry J.D., Windebank A., Staff N.P., Maragakis N.J., van den Berg L.H., Genge A., Miller R., Baloh R.H., Kern R. (2020). Addressing heterogeneity in amyotrophic lateral sclerosis CLINICAL TRIALS. Muscle Nerve.

[5] Gros-Louis F., Gaspar C., Rouleau G.A. (2006). Genetics of familial and sporadic amyotrophic lateral sclerosis. Biochim. Biophys. Acta.

[6] Rosen D.R., Siddique T., Patterson D., Figlewicz D.A., Sapp P., Hentati A., Donaldson D., Goto J., O′Regan J.P., Deng H.X. (1993). Mutations in Cu/Zn superoxide dismutase gene are associated with familial amyotrophic lateral sclerosis. Nature.

[7] Kwiatkowski T.J., Bosco D.A., Leclerc A.L., Tamrazian E., Vanderburg C.R., Russ C., Davis A., Gilchrist J., Kasarskis E.J., Munsat T. (2009). Mutations in the FUS/TLS gene on chromosome 16 cause familial amyotrophic lateral sclerosis. Science.

[8] Sreedharan J., Blair I.P., Tripathi V.B., Hu X., Vance C., Rogelj B., Ackerley S., Durnall J.C., Williams K.L., Buratti E. (2008). TDP-43 mutations in familial and sporadic amyotrophic lateral sclerosis. Science.

[9] Byrne S., Elamin M., Bede P., Shatunov A., Walsh C., Corr B., Heverin M., Jordan N., Kenna K., Lynch C. (2012). Cognitive and clinical characteristics of patients with amyotrophic lateral sclerosis carrying a C9orf72 repeat expansion: A population-based cohort study. Lancet Neurol..

[10] Kawahara Y., Mieda-Sato A. (2012). TDP-43 promotes microRNA biogenesis as a component of the Drosha and Dicer complexes. Proc. Natl. Acad. Sci. USA.

[11] Brooks B.R., Miller R.G., Swash M., Munsat T.L., World Federation of Neurology Research Group on Motor Neuron Diseases (2000). El Escorial revisited: Revised criteria for the diagnosis of amyotrophic lateral sclerosis. Amyotroph. Lateral Scler. Other Mot. Neuron Disord..

[12] Carvalho M.D., Swash M. (2009). Awaji diagnostic algorithm increases sensitivity of El Escorial criteria for ALS diagnosis. Amyotroph. Lateral Scler..

[13] Hannaford A., Pavey N., van den Bos M., Geevasinga N., Menon P., Shefner J.M., Kiernan M.C., Vucic S. (2021). Diagnostic Utility of Gold Coast Criteria in Amyotrophic Lateral Sclerosis. Ann. Neurol..

[14] Sturmey E., Malaspina A. (2022). Blood biomarkers in ALS: Challenges, applications and novel frontiers. Acta Neurol. Scand..

[15] Benatar M., Granit V., Andersen P.M., Grignon A.L., McHutchison C., Cosentino S., Malaspina A., Wuu J. (2022). Mild motor impairment as prodromal state in amyotrophic lateral sclerosis: A new diagnostic entity. Brain.

[16] Roy B., Lee E., Li T., Rampersaud M. (2022). Role of miRNAs in Neurodegeneration: From Disease Cause to Tools of Biomarker Discovery and Therapeutics. Genes.

[17] Gentile G., Morello G., La Cognata V., Guarnaccia M., Conforti F.L., Cavallaro S. (2022). Dysregulated miRNAs as Biomarkers and Therapeutical Targets in Neurodegenerative Diseases. J. Pers. Med..

[18] Panio A., Cava C., D′Antona S., Bertoli G., Porro D. (2022). Diagnostic Circulating miRNAs in Sporadic Amyotrophic Lateral Sclerosis. Front. Med..

[19] Lu J., Getz G., Miska E.A., Alvarez-Saavedra E., Lamb J., Peck D., Sweet-Cordero A., Ebert B.L., Mak R.H., Ferrando A.A. (2005). MicroRNA expression profiles classify human cancers. Nature.

[20] Weber J.A., Baxter D.H., Zhang S., Huang D.Y., Huang K.H., Lee M.J., Galas D.J., Wang K. (2010). The microRNA spectrum in 12 body fluids. Clin. Chem..

[21] Ruffo P., Strafella C., Cascella R., Caputo V., Conforti F.L., Ando S., Giardina E. (2021). Deregulation of ncRNA in Neurodegenerative Disease: Focus on circRNA, lncRNA and miRNA in Amyotrophic Lateral Sclerosis. Front. Genet..

[22] Conforti F.L., Sproviero W., Simone I.L., Mazzei R., Valentino P., Ungaro C., Magariello A., Patitucci A., La Bella V., Sprovieri T. (2011). TARDBP gene mutations in south Italian patients with amyotrophic lateral sclerosis. J. Neurol. Neurosurg. Psychiatry.

[23] Czell D., Andersen P.M., Morita M., Neuwirth C., Perren F., Weber M. (2013). Phenotypes in Swiss patients with familial ALS carrying TARDBP mutations. Neurodegener. Dis..

[24] Mitsuzawa S., Akiyama T., Nishiyama A., Suzuki N., Kato M., Warita H., Izumi R., Osana S., Koyama S., Kato T. (2018). TARDBP p.G376D mutation, found in rapid progressive familial ALS, induces mislocalization of TDP-43. eNeurologicalSci.

[25] Pfaffl M.W. (2001). A new mathematical model for relative quantification in real-time RT-PCR. Nucleic Acids Res..

[26] Ingre C., Roos P.M., Piehl F., Kamel F., Fang F. (2015). Risk factors for amyotrophic lateral sclerosis. Clin. Epidemiol..

[27] Buratti E., De Conti L., Stuani C., Romano M., Baralle M., Baralle F. (2010). Nuclear factor TDP-43 can affect selected microRNA levels. FEBS J..

[28] Freischmidt A., Muller K., Ludolph A.C., Weishaupt J.H. (2013). Systemic dysregulation of TDP-43 binding microRNAs in amyotrophic lateral sclerosis. Acta Neuropathol. Commun..

[29] Karagkouni D., Paraskevopoulou M.D., Chatzopoulos S., Vlachos I.S., Tastsoglou S., Kanellos I., Papadimitriou D., Kavakiotis I., Maniou S., Skoufos G. (2018). DIANA-TarBase v8: A decade-long collection of experimentally supported miRNA-gene interactions. Nucleic Acids Res..

[30] Freischmidt A., Muller K., Zondler L., Weydt P., Volk A.E., Bozic A.L., Walter M., Bonin M., Mayer B., von Arnim C.A. (2014). Serum microRNAs in patients with genetic amyotrophic lateral sclerosis and pre-manifest mutation carriers. Brain.

[31] Freischmidt A., Muller K., Zondler L., Weydt P., Mayer B., von Arnim C.A., Hubers A., Dorst J., Otto M., Holzmann K. (2015). Serum microRNAs in sporadic amyotrophic lateral sclerosis. Neurobiol. Aging.

[32] Wanet A., Tacheny A., Arnould T., Renard P. (2012). miR-212/132 expression and functions: Within and beyond the neuronal compartment. Nucleic Acids Res..

[33] Lewis B.P., Burge C.B., Bartel D.P. (2005). Conserved seed pairing, often flanked by adenosines, indicates that thousands of human genes are microRNA targets. Cell.

[34] Vacchiano V., Mastrangelo A., Zenesini C., Masullo M., Quadalti C., Avoni P., Polischi B., Cherici A., Capellari S., Salvi F. (2021). Plasma and CSF Neurofilament Light Chain in Amyotrophic Lateral Sclerosis: A Cross-Sectional and Longitudinal Study. Front. Aging Neurosci..

[35] Poesen K., Van Damme P. (2018). Diagnostic and Prognostic Performance of Neurofilaments in ALS. Front. Neurol..

[36] Morlando M., Dini Modigliani S., Torrelli G., Rosa A., Di Carlo V., Caffarelli E., Bozzoni I. (2012). FUS stimulates microRNA biogenesis by facilitating co-transcriptional Drosha recruitment. EMBO J..

[37] Waller R., Wyles M., Heath P.R., Kazoka M., Wollff H., Shaw P.J., Kirby J. (2017). Small RNA Sequencing of Sporadic Amyotrophic Lateral Sclerosis Cerebrospinal Fluid Reveals Differentially Expressed miRNAs Related to Neural and Glial Activity. Front. Neurosci..

[38] Lagos-Quintana M., Rauhut R., Yalcin A., Meyer J., Lendeckel W., Tuschl T. (2002). Identification of tissue-specific microRNAs from mouse. Curr. Biol..

[39] Sim S.E., Lim C.S., Kim J.I., Seo D., Chun H., Yu N.K., Lee J., Kang S.J., Ko H.G., Choi J.H. (2016). The Brain-Enriched MicroRNA miR-9-3p Regulates Synaptic Plasticity and Memory. J. Neurosci..

[40] Wang X., Wang X. (2006). Systematic identification of microRNA functions by combining target prediction and expression profiling. Nucleic Acids Res..

[41] Han D., Dong X., Zheng D., Nao J. (2019). MiR-124 and the Underlying Therapeutic Promise of Neurodegenerative Disorders. Front. Pharmacol..

[42] Vaz A.R., Vizinha D., Morais H., Colaco A.R., Loch-Neckel G., Barbosa M., Brites D. (2021). Overexpression of miR-124 in Motor Neurons Plays a Key Role in ALS Pathological Processes. Int. J. Mol. Sci..

[43] Matamala J.M., Arias-Carrasco R., Sanchez C., Uhrig M., Bargsted L., Matus S., Maracaja-Coutinho V., Abarzua S., van Zundert B., Verdugo R. (2018). Genome-wide circulating microRNA expression profiling reveals potential biomarkers for amyotrophic lateral sclerosis. Neurobiol. Aging.

[44] Zhang Z., Pi J., Zou D., Wang X., Xu J., Yu S., Zhang T., Li F., Zhang X., Zhao H. (2019). microRNA arm-imbalance in part from complementary targets mediated decay promotes gastric cancer progression. Nat. Commun..

[45] Zhang S., Zhang R., Xu R., Shang J., He H., Yang Q. (2020). MicroRNA-574-5p in gastric cancer cells promotes angiogenesis by targeting protein tyrosine phosphatase non-receptor type 3 (PTPN3). Gene.

[46] Mackenzie I.R., Bigio E.H., Ince P.G., Geser F., Neumann M., Cairns N.J., Kwong L.K., Forman M.S., Ravits J., Stewart H. (2007). Pathological TDP-43 distinguishes sporadic amyotrophic lateral sclerosis from amyotrophic lateral sclerosis with SOD1 mutations. Ann. Neurol..

[47] Boudreau R.L., Jiang P., Gilmore B.L., Spengler R.M., Tirabassi R., Nelson J.A., Ross C.A., Xing Y., Davidson B.L. (2014). Transcriptome-wide discovery of microRNA binding sites in human brain. Neuron..

