# Supplementary material for: Deregulation of Plasma microRNA Expression in a TARDBP-ALS Family
Source: Biomolecules. 2023 Apr 21;13(4):706. doi: 10.3390/biom13040706 (PMC10135769; doi:10.3390/biom13040706)
Supplement: Supplementary file 1 [file biomolecules-13-00706-s001.zip › biomolecules-2313726-supplementary-proofdone - revised/Supplementary Files/Supplementary Figure 1.docx]

**Supplementary Figure 1.** Serum miRNA deregulation values of one sample compared to affected subjects from the same family.


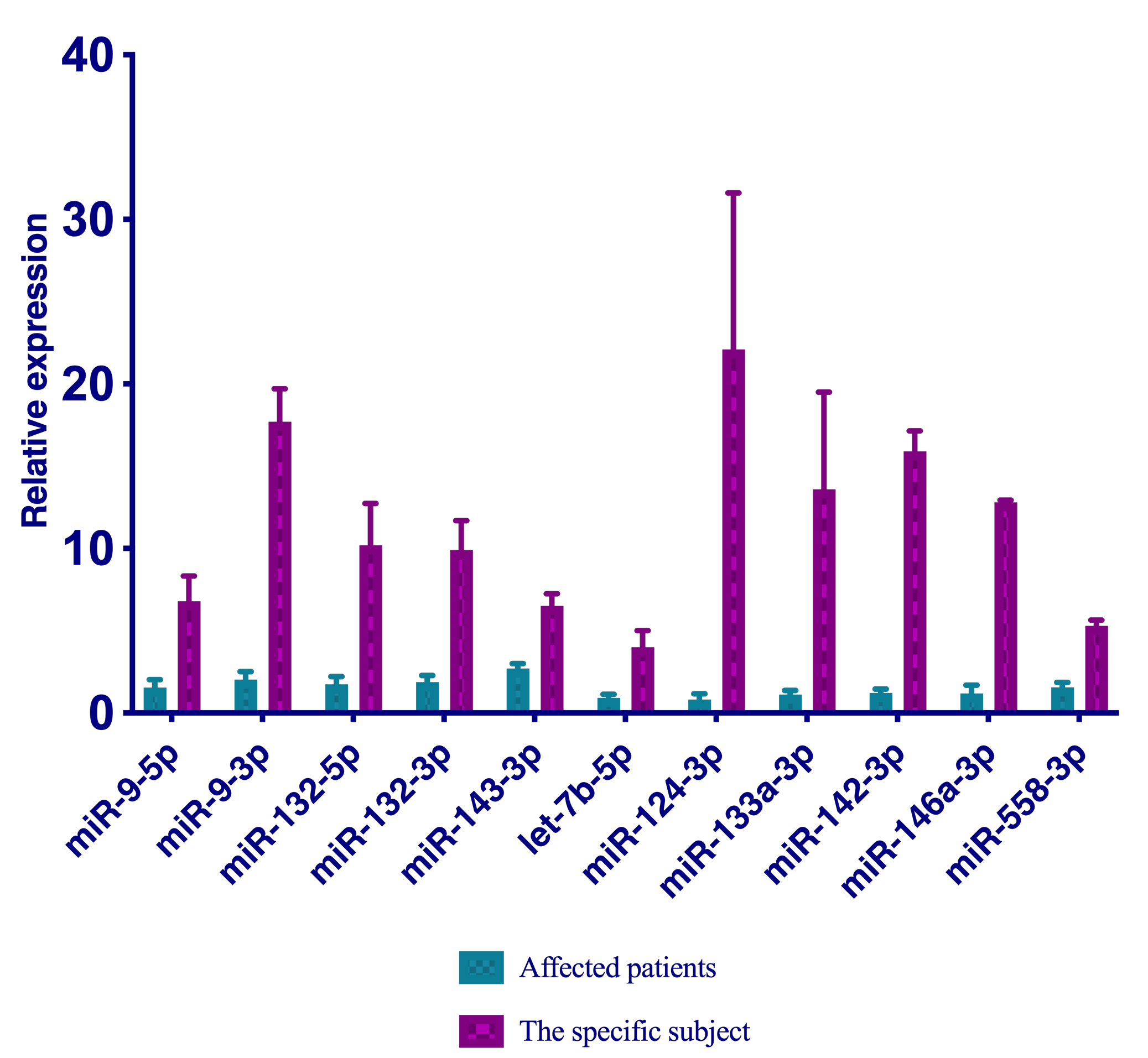


Relative expression of miRNA in plasma measured with qPCR. Normalization was performed relative to the spiked-in C. elegans miRNA (Cel-miR-39-3p) and two endogenous miRNAs (hsa-miR-191-5p and hsa-miR-93-5p).
