# Supplementary material for: Deregulation of Plasma microRNA Expression in a TARDBP-ALS Family
Source: Biomolecules. 2023 Apr 21;13(4):706. doi: 10.3390/biom13040706 (PMC10135769; doi:10.3390/biom13040706)
Supplement: Supplementary file 1 [file biomolecules-13-00706-s001.zip › biomolecules-2313726-supplementary-proofdone - revised/Supplementary Files/Supplementary Table 1.docx]

### Supplementary Table 1. List of miRNAs analyzed with relative assay identification (ID) and mature miRNA sequence.

| Assay Name | Assay ID | Mature miRNA Sequence |
| --- | --- | --- |
| hsa-miR-9-5p | 478214_mir | UCUUUGGUUAUCUAGCUGUAUGA |
| hsa-miR-132-5p | 478705_mir | ACCGUGGCUUUCGAUUGUUACU |
| hsa-miR-132-3p | 477900_mir | UAACAGUCUACAGCCAUGGUCG |
| hsa-miR-143-5p | 478713_mir | GGUGCAGUGCUGCAUCUCUGGU |
| hsa-miR-143-3p | 477912_mir | UGAGAUGAAGCACUGUAGCUC |
| hsa-miR-574-5p | 479357_mir | UGAGUGUGUGUGUGUGAGUGUGU |
| hsa-miR-574-3p | 478163_mir | CACGCUCAUGCACACACCCACA |
| hsa-miR-558-3p | 479044_mir | UGAGCUGCUGUACCAAAAU |
| hsa-miR-663a | 479445_mir | AGGCGGGGCGCCGCGGGACCGC |
| hsa-let-7b-5p | 478576_mir | UGAGGUAGUAGGUUGUGUGGUU |
| hsa-miR-9-3p | 478211_mir | AUAAAGCUAGAUAACCGAAAGU |
| hsa-miR-124-3p | 480901_mir | UAAGGCACGCGGUGAAUGCCAA |
| hsa-miR-133a-3p | 478511_mir | UUUGGUCCCCUUCAACCAGCUG |
| hsa-miR-133b | 480871_mir | UUUGGUCCCCUUCAACCAGCUA |
| hsa-miR-142-3p | 477910_mir | UGUAGUGUUUCCUACUUUAUGGA |
| hsa-miR-146a-3p | 478714_mir | CCUCUGAAAUUCAGUUCUUCAG |
| hsa-miR-155-3p | 477926_mir | CUCCUACAUAUUAGCAUUAACA |
| hsa-miR-218-5p | 477977_mir | UUGUGCUUGAUCUAACCAUGU |
| hsa-miR-338-5p | 478038_mir | AACAAUAUCCUGGUGCUGAGUG |
