# Supplementary material for: Deregulation of Plasma microRNA Expression in a TARDBP-ALS Family
Source: Biomolecules. 2023 Apr 21;13(4):706. doi: 10.3390/biom13040706 (PMC10135769; doi:10.3390/biom13040706)
Supplement: Supplementary file 1 [file biomolecules-13-00706-s001.zip › biomolecules-2313726-supplementary-proofdone - revised/Supplementary Files/Supplementary Table 2.docx]

| miRNA name | Gene Target | Method | Tissue | Cell Lines | Prediction Score | Targeted region | Binding Type | Publications |
| --- | --- | --- | --- | --- | --- | --- | --- | --- |
| hsa-miR-124-3p | *TARDBP* | High  throughput | Liver | HEPG2 | / | / | / | [40] |
| hsa-miR-132-3p | *TARDBP* | High  throughput | Kidney | 293S | 0.522 | 3’ UTR | 6mer | [33] |
| hsa-miR-218-5p | *TARDBP* | High  throughput | Kidney | HEK293 | / | / | / | [46] |
|  |  |  | Brain | / | / | / | / | [47] |

**Supplementary Table 2.** List of miRNAs targeting *TARDBP*.
