# Supplementary material for: Deregulation of Plasma microRNA Expression in a TARDBP-ALS Family
Source: Biomolecules. 2023 Apr 21;13(4):706. doi: 10.3390/biom13040706 (PMC10135769; doi:10.3390/biom13040706)
Supplement: Supplementary file 1 [file biomolecules-13-00706-s001.zip › biomolecules-2313726-supplementary-proofdone - revised/Supplementary Files/Supplementary Table 3.docx]

**Supplementary Table 3.** List of serum miRNA values for one specific affected patient normalized against one exogenous (cel-miR-39) and two endogenous (hsa-miR-191-5p and hsa-miR-93-5p) controls.

| **miRNA name** | **Fold change (mean ± SD)** |
| --- | --- |
| hsa-miR-9-5p | 6,8 ± 1,53 |
| hsa-miR-132-5p | 10,2 ± 2,54 |
| hsa-miR-132-3p | 9,9 ± 1,80 |
| hsa-miR-143-3p | 6,5 ± 0,75 |
| hsa-miR-558-3p | 5,3 ± 0,35 |
| hsa-let-7b-5p | 4,0 ± 1,0 |
| hsa-miR-9-3p | 17,7 ± 2,0 |
| hsa-miR-124-3p | 66,2 ± 28,7 |
| hsa-miR-133a-3p | 40,8 ± 17,7 |
| hsa-miR-133b | too high |
| hsa-miR-142-3p | 15,9 ± 1,24 |
| hsa-miR-146a-3p | 12,8 ± 0,13 |
